# Supplementary material for: Kinetics-based inference of environment-dependent microbial interactions and their dynamic variation
Source: mSystems. 2024 Apr 29;9(5):e01305-23. doi: 10.1128/msystems.01305-23 (PMC11097648; doi:10.1128/msystems.01305-23)
Supplement: Supplemental material — Supplemental text; Tables S1 and S2; Fig. S1 to S4. [file msystems.01305-23-s0001.pdf]

SUPPLEMENTARY TEXT:

Connection between KIDI and MacArthur's Consumer-Resource Model

In this supplementary text, we illustrate how KIDI can be extended to traditional ecological models, such as MacArthur's consumer-resource (C-R) model, by elucidating their interconnection. MacArthur (1970) formulated the per-capita growth rate of consumer species i (X_i) and resource species j (R_j) as follows:

$$\frac{1}{X_i}\frac{dX_i}{dt} = b_i\left(\sum_{k=1}^{n} c_{ik}w_kR_k - m_i\right) \qquad \text{(A1)}$$

$$\frac{1}{R_k}\frac{dR_k}{dt} = r_k\left(1 - \frac{R_k}{K_k}\right) - \sum_{i=1}^{n} c_{ik}X_i \qquad \text{(A2)}$$

where

- X_i is a population density of consumer specie i
- R_k is the population density of resource species k
- w_k is the value of one unit of resource species j to the consumer
- c_{ik} is the rate at which consumer species i captures resource j per unit abundance of resource species j
- m_i is, the maintenance term (i.e., the total value of resource that must be harvested per capita for maintaining the current population density of consumer species i
- b_i is a factor converting the resource excess into the per-capita growth rate
- r_k is per-capita growth rate of resource species j
- K_k is carrying capacity for resource species j

MacArthur assumed faster dynamics of resource populations compared to consumer populations (i.e., r_k and c_{ik} are much larger than $b_ic_{ik}w_k$ and b_im_i) so that Eq. (A2) is reduced to an algebraic equation (by setting $\frac{dR_j}{dt} = 0$). By substituting R_k in Eq. (A1) for X_i's, MacArthur derived a'_{ij} (i.e., the competitive relationship between the two consumers i and j, or more precisely the impact of consumer j on the growth on consumer i) in the Lotka-Volterra (LV) model as follows:

$$a'_{ij} = \sum_{k=1}^{n} \frac{c_{ik}c_{jk}w_kK_k}{r_k} \qquad \text{(A3)}$$

where we use the prime notation in a'_{ij} to differentiate it from the definition of a_{ij} commonly used in a generalized LV model. These two notations are related by $-b_ia'_{ij} = a_{ij}$.

We now show that the above equation can be derived by KIDI. Interspecies interaction coefficient a_{ij} (not a'_{ij}) for the system with multiple resources in KIDI are defined as follows:

$$a_{ij} = \sum_{k=1}^{n} \left(\frac{\partial \mu_i}{\partial R_k}\right)\left(\frac{\partial R_k}{\partial X_j}\right) \qquad \text{(A4)}$$

where μ_i is the specific (or per-capita) growth rate of species i (i.e., $\frac{1}{X_i}\frac{dX_i}{dt}$). The first term $\left(\frac{\partial \mu_i}{\partial R_k}\right)$ is obtained simply by taking the derivative of the per-capita growth rate for X_i with respect to R_k in Eq. (A1), i.e.,

$$\left(\frac{\partial \mu_i}{\partial R_k}\right) = b_i c_{ik} w_k \qquad \text{(A5)}$$

Next, we take the derivative of the steady-state equation of Eq. (A2) with respect to X_j to give the second term $\left(\frac{\partial R_k}{\partial X_j}\right)$ as follows:

$$\left(\frac{\partial R_k}{\partial X_j}\right) = -\frac{K_k c_{jk}}{r_k} \qquad \text{(A6)}$$

With the substitutions using Eqs. (A5) and (A6), the final form of a_{ij} becomes

$$a_{ij} = -b_i \sum_{k=1}^{n} c_{ik} w_k \frac{K_k c_{ij}}{r_k} \qquad \text{(A7)}$$

The above equation proves $a_{ij} = -b_i a'_{ij}$.

SUPPLEMENTARY TABLES AND FIGURES

Table S1. Initial culture conditions for experimental data displayed in Fig. 2.

Exp #	Corresponding figures	Initial inoculum size (OD)	Glucose (g/l)	Tryptophan (mg/l)	Tyrosine (mg/l)	# of FBs added	Time FBs added (hr)
1	Fig. 2A (top panel)	ΔTrp only: 0.01	5.838	10	-	-	-
2	Fig. 2A (middle panel)	ΔTrp only: 0.01	5.870	20	-	-	-
3	Fig. 2A (bottom panel)	ΔTrp only: 0.01	5.936	40	-	-	-
4	Fig. 2B (top panel)	ΔTyr only: 0.01	5.920	-	10	-	-
5	Fig. 2B (middle panel)	ΔTyr only: 0.01	5.909	-	20	-	-
6	Fig. 2B (bottom panel)	ΔTyr only: 0.01	5.863	-	40	-	-
7	Fig. 2C (top panel)	ΔTrp: 0.01<br>ΔTyr: 0.01	3.851	0.4	0.4	-	-
8	Fig. 2C (middle panel)	ΔTrp: 0.01<br>ΔTyr: 0.01	0.626	0.4	0.4	3	7.5
9	Fig. 2C (bottom panel)	ΔTrp: 0.01<br>ΔTyr: 0.01	0.620	0.4	0.4	5	7.5
10	Fig. 2D (top panel)	ΔTrp: 0.01<br>ΔTyr: 0.01	2.878	0.4	0.4	-	-
11	Fig. 2D (middle panel)	ΔTrp: 0.01<br>ΔTyr: 0.01	0.375	0.4	0.4	3	10
12	Fig. 2D (bottom panel)	ΔTrp: 0.01<br>ΔTyr: 0.01	0.377	200	200	3	10

Table S2. Sequence of the primers used in the qPCR analysis.

**Primer**	**Sequence**
Trp F	GCCGATGCCTGCTTATTA
Trp R	GCTCCTGTTCCTCTTCAT
Tyr F	CATTATGTCGTCAGAGCG
Tyr R	CCTTGCGGAAACTGTCAA

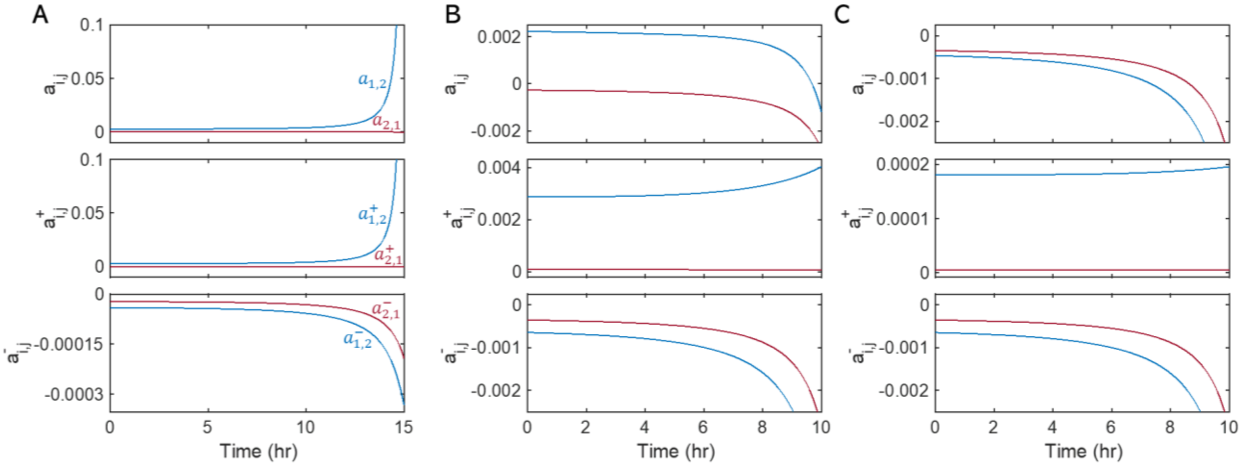

Figure S1. Zoom-in views of in interaction parameters, $a_{i,j}$, $a^{+}_{i,j}$, and $a^{-}_{i,j}$ in **Figs. 3A, 3B, and 3C,** respectively. The color scheme is the same as in **Fig. 3**.

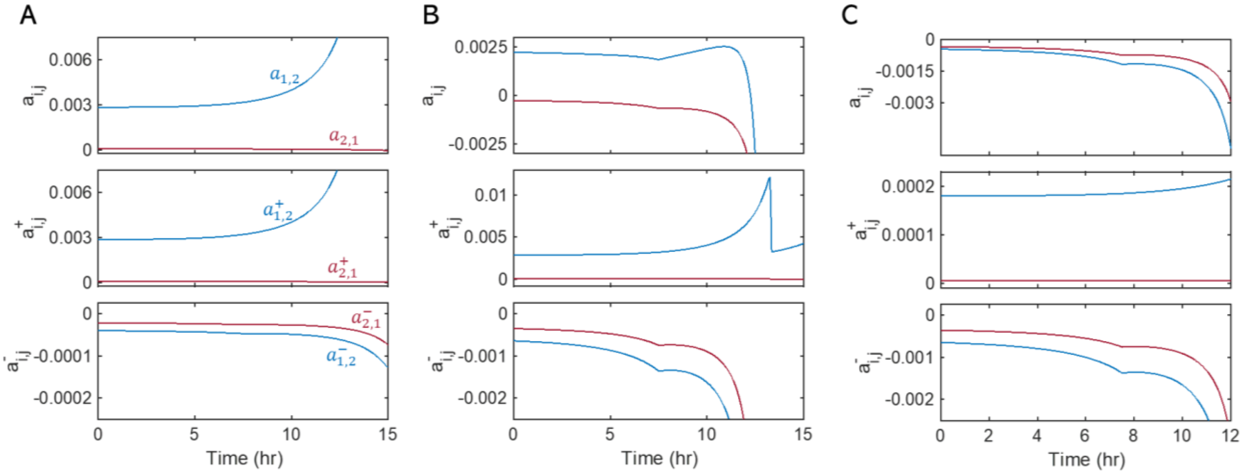

Figure S2. Zoom-in views of in interaction parameters, $a_{i,j}$, $a^+_{i,j}$, and $a^-_{i,j}$ in **Figs. 4A, 4B, and 4C,** respectively. The color scheme is the same as in **Fig. 4**.

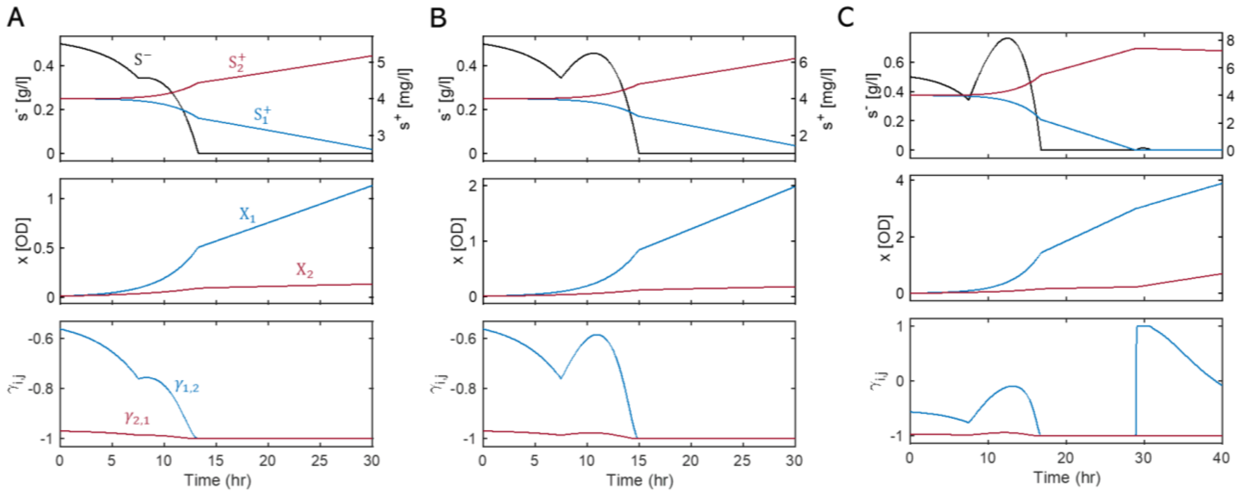

Figure S3. The predicted impacts of the number of added FBs on the substrate profile (S^-), the population dynamics (X_1 and X_2), and the interaction parameter ($\gamma_{i,j}$) in fed-batch cultures with initial substrate concentrations of 2 g/l of glucose, 0.4 mg/l of tryptophan, and 0.4 mg/l of tyrosine. The number of glucose BFs added at 7.5 hours: **A.** 3 FBs; **B.** 6 FBs; and **C.** 10 FBs.

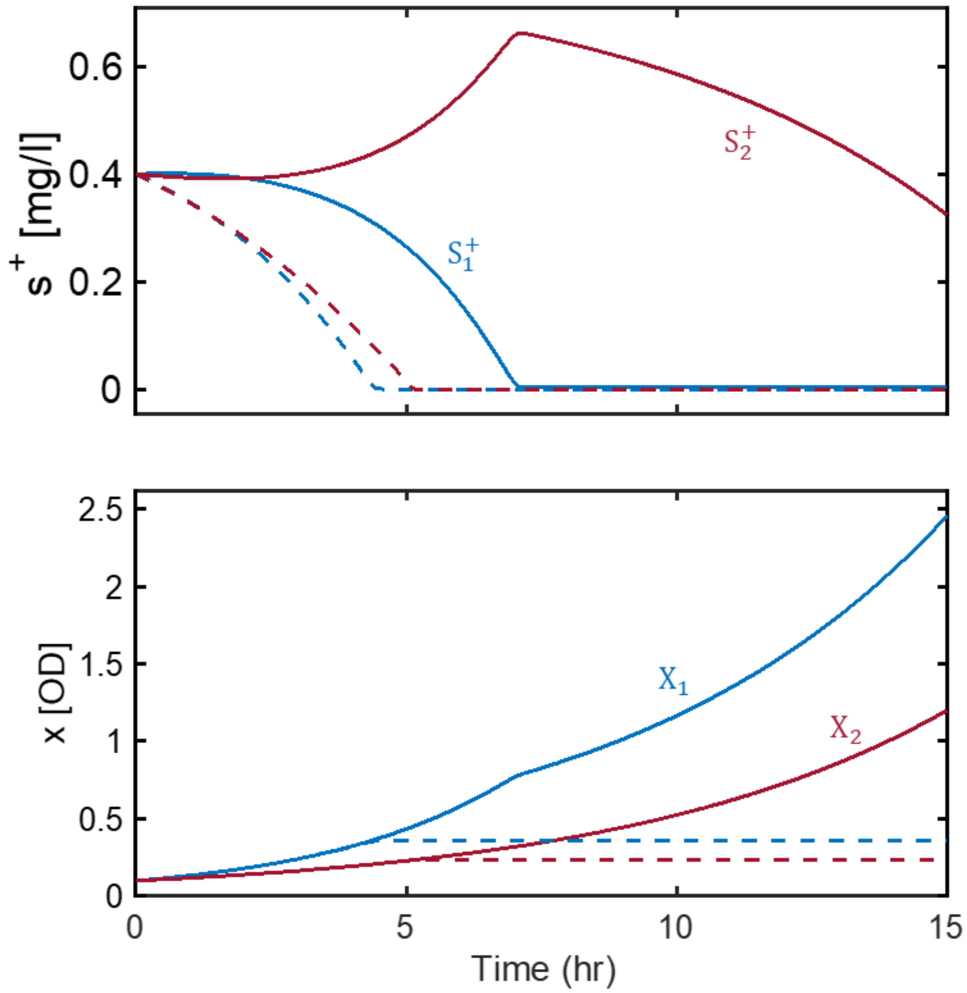

Figure S4. Simulated substrate concentrations (top), and biomass concentrations (bottom) with and without amino acid cross-feeding in the batch culture with excessive provision of glucose. Initial substrate concentrations of amino acids: 0.4 mg/l of tryptophan and 0.4 mg/l of tyrosine. Solid lines denote the case with cross-feeding; dashed lines denote the case of preventing cross-feeding in simulations.
